# Trends and controversies in carotid artery stenosis treatment

**DOI:** 10.12688/f1000research.25922.1

**Published:** 2020-08-07

**Authors:** Rakhee Lalla, Prashant Raghavan, Seemant Chaturvedi

**Affiliations:** 1 Department of Neurology, University of Maryland School of Medicine, Baltimore, MD, USA; 2 Department of Radiology, University of Maryland School of Medicine, Baltimore, MD, USA

**Keywords:** carotid stenosis, endarterectomy, statins

## Abstract

Despite the completion of several multi-center trials, the management of carotid stenosis remains in flux. Key questions include the role of intensive medical management in the treatment of asymptomatic carotid stenosis. In addition, identification of patients with symptomatic stenosis who will most benefit from carotid revascularization remains a priority. The role of newer imaging techniques such as carotid plaque analysis with magnetic resonance imaging is also challenging current treatment paradigms. These topics are explored in this topical update.

## Introduction

Extracranial internal carotid artery (ICA) stenosis is a leading cause of ischemic stroke, accounting for about 10% of strokes. Despite the completion of several randomized controlled trials to evaluate the efficacy of surgical or endovascular intervention for ICA stenosis, there are still shortcomings in our modern database. These shortcomings include the failure to study intensive medical therapy (IMT) for ICA stenosis. In addition, there is pronounced international variation with regard to the use of carotid endarterectomy (CEA) for asymptomatic stenosis. Finally, newer imaging techniques may provide a new window into stroke risk stratification. These topics will be addressed in this review.

## Asymptomatic carotid stenosis

Before discussion of recent study results, it is important to define the terms “asymptomatic” and “symptomatic”. Symptomatic carotid stenosis refers to patients with a stroke or transient ischemic attack (TIA) in the previous 6 months which is related to the stenosis. Asymptomatic stenosis includes patients who have never had ischemic symptoms or patients with symptoms more than 6 months ago. In addition, non-localizing symptoms such as “dizziness” or “lightheadedness” are not considered symptomatic. Nor is syncope considered a symptom of unilateral ICA stenosis. Patients with no clear symptoms but abnormalities on brain imaging studies are considered to have “silent strokes” but are not considered “symptomatic” according to recent clinical trials.

Medical therapy for the management of ischemic stroke has greatly evolved over the last 30 years. This has been especially evident in cases of asymptomatic carotid stenosis (ACS). The Asymptomatic Carotid Atherosclerosis Study (ACAS), conducted between 1987 and 1993, found an annual stroke risk of about 2 to 2.5% in patients with ACS of 60 to 99%. The subsequent Asymptomatic Carotid Surgery Trial (ACST) interestingly showed a decline in annual stroke risk from 1.1 to 0.7% in the latter half of the study, as improvements in medical therapy became key factors in the reduction of vascular risk. Use of lipid-lowering therapy increased from less than 10% in 1993 to more than 80% in 2008. Both of these trials favored CEA over medical therapy alone in select patients up to age 75 with asymptomatic 60 to 99% carotid disease. Key points regarding these early trials include the following:

• All patients recruited into ACAS, ACST, and the Veterans Affairs cooperative study were considered to be at low or average risk of complications from CEA.

• The medical treatment used in these trials was rudimentary by today’s standards. It generally consisted of prescribing aspirin and some advice on risk factors such as hypertension and cigarette smoking.

• Overall, 1.7 to 4.5% of patients who had CEA in these trials had a stroke or died within 30 days of CEA. The overall 1-month preoperative stroke or death rate in ACST was 3.1%.

The benefit of surgical intervention, however, has been questioned in recent years as aggressive medical management of vascular risk factors continues to improve, including the more widespread use of antiplatelet agents, lipid-lowering therapy, antihypertensives, and improved glycemic control. The SMART study, for example, found an annual overall ischemic stroke risk of less than 1% in a population with moderate and severe ACS treated medically, and further subgroup analysis revealed no difference in the percentage of strokes attributed to large-vessel disease ^1^. Given the diminishing stroke incidence with use of best medical therapy, further trials are necessary to determine the role of surgical intervention. Reductions in stroke/death following CEA and CAS also support performance of new trials. The ongoing CREST-2 trial seeks to answer this question, focusing on patients with severe stenosis (70–99%), and is comparing intensive medical management alone versus surgical intervention (CEA or stenting) in addition to IMT ^2^.

Despite controversy and misconceptions regarding the adequacy of best medical intervention alone in the management of ACS, CEA and stenting remain part of the current treatment algorithm. However, the American Heart Association (AHA), American Stroke Association, and European Society for Vascular Surgery recommend an assessment of predicted life expectancy prior to a decision on surgical intervention. An important consideration for the clinician is patient life expectancy. The Society for Vascular Surgery guidelines further specify that patients with ACS of greater than 60% should be considered for surgical intervention if life expectancy is thought to be more than 3 years and perioperative risk of stroke or death is less than 3% ^3^. Wallaert *et al*. ^4^ report 5-year survival after CEA of 82% in a cohort of asymptomatic patients. Risk factors for mortality include increasing age, diabetes, congestive heart failure, poor renal function, lack of statin use, smoking history, chronic obstructive pulmonary disease, and contralateral ICA stenosis. On the other hand, in asymptomatic patients with moderate to severe stenosis treated with best medical therapy alone, 5-year survival was 77% and all-cause mortality remained high (4.6% annually and the majority were cardiovascular etiologies) regardless of stroke risk ^3^. The current European Society for Vascular Surgery guidelines take this into account, recommending consideration of CEA for those with asymptomatic 60 to 99% stenosis with a life expectancy of more than 5 years and at least one feature suggestive of a higher stroke risk on best medical therapy ^5^. The American Academy of Neurology guideline recommends consideration of CEA only in patients between 40 and 75 years of age ^6^. Therefore, it is crucial to stratify patients on the basis of not only life expectancy but also characteristics of their carotid disease thought to increase stroke risk, such as plaque area, echolucency, presence of intraplaque hemorrhage (IPH) or silent infarction on magnetic resonance imaging (MRI), or evidence of spontaneous embolization on transcranial Doppler (TCD). Use of these additional imaging modalities should be considered in decision making for patients with ACS and is discussed later in this article. A risk stratification tool that takes into account both a patient’s cardiovascular comorbidities and plaque characteristics would be a valuable addition to the management algorithm for this population.

There is increasing interest not only in ischemic stroke but also in the relationship between ICA stenosis and cognitive decline. In a state of chronic hypoperfusion with bilateral carotid ligation, animal models have shown evidence of worse cognitive task performance that subsequently improves with restoration of flow. This was again demonstrated in studies measuring cerebral hemodynamics in patients with severe carotid stenosis revealing a state of chronic hypoperfusion and decline in ipsilateral hemisphere cognitive ability ^7^. Several alternative mechanisms for the relationship of carotid stenosis and cognitive decline, including silent artery to artery emboli or simply uncontrolled vascular risk factors contributing to worsening small-vessel disease, have been proposed ^5^. However, studies using TCD emboli detection argue against silent embolic infarcts; a Manchester (UK) series reported the presence of microemboli in only 2% of patients with severe ACS and dementia ^5^. According to more recent literature, there seems to be a relationship between the hemodynamic effects of stenosis and cognitive decline, supporting the theory of chronic hypoperfusion and impaired cerebral vasoreactivity ^8^. Presumably, flow restoration with surgical intervention should improve performance on cognitive tasks but the evidence for this has been variable. The ongoing CREST-H trial, a subset of CREST-2, plans to assess changes in cognition annually after both medical and surgical interventions, which will shed light on the utility of surgery for the purpose of improving cognitive function ^9^. Until conclusive study results are available, carotid revascularization is not recommended as a treatment for dementia.

## Symptomatic carotid stenosis

In general, more widespread use of preventative therapies has been associated with a decline in the rate of stroke over the past five decades. A systematic review of secondary prevention studies focused on stroke between 1960 and 2009 and found significant declines in the rate of total stroke (43% decrease), fatal stroke, and major vascular events (including myocardial infarction and vascular death) ^10^. The authors found that increasing use of antithrombotic medications and lower achieved blood pressure were two of the main contributors to the decline in stroke recurrence rates ^10^. Statins have also been found to be useful for stroke prevention in several settings, including for primary prevention and secondary prevention. The recently reported Treat Stroke to Target study found that a lower low-density lipoprotein (LDL) target (<70 mg/dL versus <100 mg/dL) was associated with a reduced rate of stroke ^11^. Lifestyle modification is routinely recommended in professional guidelines for vascular disease prevention. For example, AHA secondary prevention guidelines recommend three or four sessions per week of moderate/vigorous aerobic exercise. In the EXPRESS study, use of multi-modality therapy (including antiplatelet therapy, statins, and antihypertensives) was found to reduce the 90-day rate of stroke following a TIA by 80% (from 10 to 2%). The regimen included dual antiplatelet therapy for 30 days, followed by monotherapy ^12^.

A multifaceted approach for vascular disease prevention has been found to be associated with a decreased rate of stroke and major vascular events. In REGARDS, levels of the AHA “Life’s simple 7 (LS7)” components (blood pressure, cholesterol, glucose, body mass index, smoking, physical activity, and diet) were each coded as poor (0 point), intermediate (1 point), or ideal (2 points) health, providing a score from 0 (poor on all domains) to 14 (ideal on all domains). Among 22,914 participants, stroke risk was decreased by 8% (95% confidence interval [CI] 5–12%) for each point increase in the LS7 ^13^. Furthermore, in the Northern Manhattan Study, increased adherence to the LS7 metric was related to a graded reduction in stroke, and a risk reduction of 59% was reported in patients with the highest adherence ^14^. These studies illustrate the value of multifaceted medical preventative therapy in the community setting.

Four key components of IMT are antithrombotic therapy, statins, blood pressure control, and lifestyle modification. The combination of these four aspects of IMT has been found to be useful for reduction of stroke in patients with large-vessel cerebral atherosclerotic disease. The Stenting and Aggressive Medical Management for Preventing Recurrent Stroke in Intracranial Stenosis (SAMMPRIS) trial compared IMT alone with IMT plus intracranial stenting in patients with severe (70–99%) narrowing of the major intracranial arteries ^15^. The primary endpoint was stroke or death within 30 days after enrollment (or after a revascularization procedure beyond 30 days) or stroke in the territory of the qualifying artery beyond 30 days.

In the SAMMPRIS trial, IMT was very effective in reaching treatment targets. The mean blood pressure in patients 4 months after study entry was 134/77. The mean LDL value 4 months after study entry was 74.4 mg/dL. The rate of smoking decreased by 7 to 10% (in absolute terms) in the two treatment groups. Moderate or vigorous exercise increased by 22 to 27% (in absolute terms) in the two treatment groups. The study was stopped prematurely since IMT alone was superior to IMT plus intracranial stenting. At 1 year, the rates of the primary endpoint were 20.0% with IMT plus stenting and 12.2% in the medical management group ( *P* = 0.009). Turan *et al*. also demonstrated the value of regular physical activity in the SAMMPRIS trial ^16^. Those patients with the most physical activity had a 40% reduction in major vascular events ^16^. This study illustrates the potent stroke prevention results that can be achieved if intensive medical management is pursued.

Multi-modality therapy has proven to be useful for patients with symptomatic carotid stenosis as well. In a study from Denmark, 115 patients received a regimen of dual antiplatelet therapy (aspirin plus clopidogrel) and simvastatin prior to CEA ^17^. After implementation of this protocol, there were no recurrent strokes prior to CEA. The frequency of neurological events prior to CEA declined from 29 to 2.5% after implementation of a “best medical therapy” protocol. A study from the UK also found that in patients with symptomatic carotid stenosis, dual antiplatelet therapy reduced the number of brain microemboli detected with TCD ^18^. This study also found that 0 out of 100 patients awaiting CEA experience a recurrent stroke while awaiting revascularization.

The medical treatment paradigm at the time of the North American Symptomatic Carotid Endarterectomy Trial (NASCET) and European Carotid Surgery Trial (ECST) compared with modern therapy is strikingly different. The different approaches are outlined in Table 1.

**Table 1.** Evolution of medical therapy for symptomatic carotid artery stenosis.

Condition	Treatment in first-generation trials	Modern treatment
Antithrombotic therapy	Aspirin alone	Aspirin plus clopidogrel
Lipids	Little statin use	High-potency statins
Blood pressure	No specific target	Systolic blood pressure less than 130 mm Hg
Smoking cessation	No pharmacologic therapy	New pharmacologic treatments
Physical activity	No specific target	Benefits understood for regular physical activity (three or four sessions of aerobic exercise per week)
Diabetes	No specific medications for cardiovascular (CV) risk	Pharmacologic treatments that reduce CV risk, hemoglobin A1C target of less than 7
High triglyceride levels	No specific treatment	Icosapent ethyl

These recent studies question the pooled subgroup analyses of NASCET and ECST. One subgroup analysis showed that the greatest benefit of CEA occurred in patients who were enrolled within 14 days of their last symptomatic event ^19^. However, the median delay from the last symptomatic event to enrollment in NASCET and ECST was more than 30 days. The new emergency management paradigm of TIA/minor stroke in TIA/stroke clinics and urgent IMT worldwide is very different from that of the old trials. It is not clear whether the immediate application of IMT soon after a TIA or minor stroke prevents future stroke or delays later strokes. However, modern registries suggest a dramatic decrease in vascular events with multifaceted medical therapy.

Given that NASCET and ECST are close to 30 years old and that modern IMT is widespread, it makes sense to revisit the question of which symptomatic patients require CEA. Patients identified as lower-risk in NASCET, such as patients with retinal events only, women, and those with the last symptomatic event more than 2 weeks previously, could fare well with IMT alone. In addition, patients with radiologic features of low risk (absence of microemboli or absence of IPH) could have acceptably low rates of stroke with IMT. Multi-center studies to assess the stroke rate in “symptomatic, lower-risk” carotid stenosis are needed. The European Carotid Surgery Trial 2 ( www.ecst2.com) includes symptomatic, lower-risk patients and studies in North America will be valuable as well.

## Carotid plaque imaging

First-generation trials such as NASCET relied on conventional angiography to define the degree of carotid stenosis. At present, non-invasive imaging methods have proliferated and newer imaging techniques can also provide prognostic information.

In broad terms, the role of imaging in carotid artery atherosclerotic disease is twofold. The first, quantification of the degree of carotid stenosis, can be accomplished to a high degree of accuracy by conventional computed tomography (CT) or MR angiography and ultrasound (US). The second, characterization of plaque morphology, is best achieved by MRI, although CT, US, and positron emission tomography (PET)-based molecular imaging techniques can provide useful information. Refinement of these techniques directed toward clearer visualization of the arterial wall has stemmed from the realization that recognizing features of plaque vulnerability, in addition to quantifying the degree of stenosis, can provide more accurate prediction of ischemic events and help guide response to therapy ^20^.

### Magnetic resonance imaging

**Figure 1.**
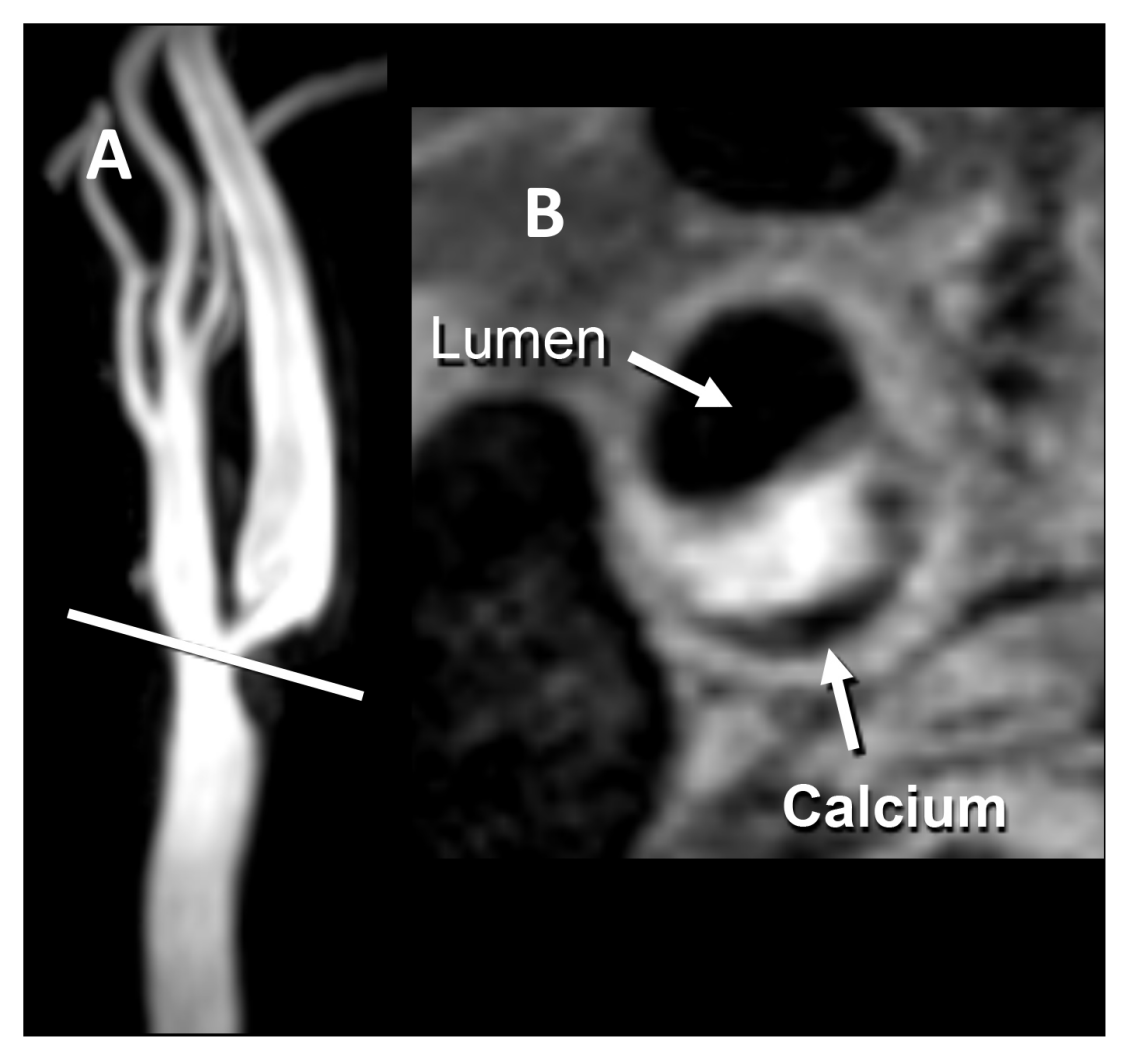
Intraplaque hemorrhage. ( **A**) Time-of-flight magnetic resonance angiography shows roughly 70% stenosis due to a plaque with intraplaque hemorrhage (IPH). IPH is seen as an area of high signal intensity on the non-contrast T1-weighted double inversion recovery image, obtained at a resolution of 0.35 × 0.35 × 2 mm. ( **B**) A low-signal-intensity area of peripheral calcification is also present. Images courtesy of Bruce Wasserman, Johns Hopkins University.

**Figure 2.**
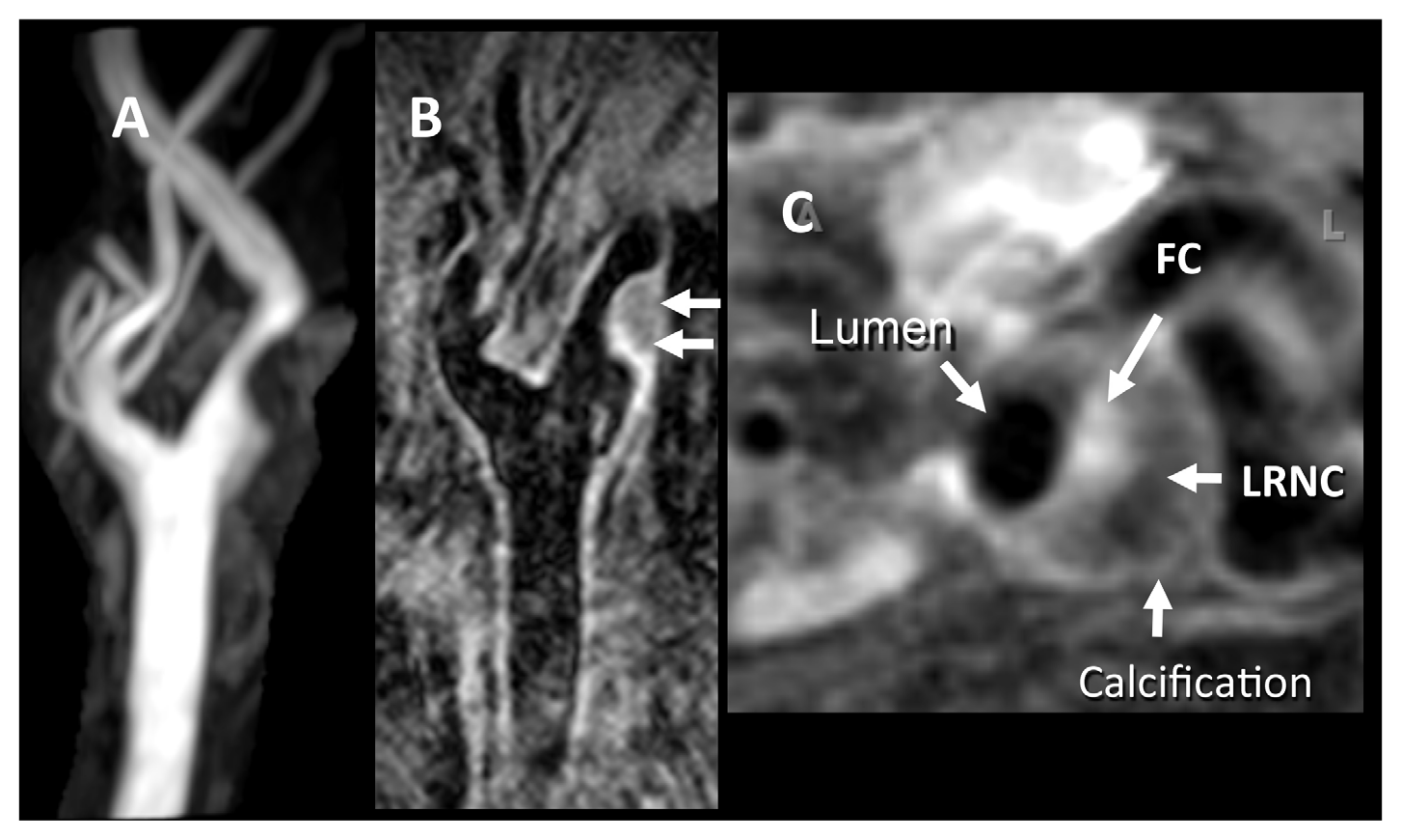
Features of complex plaque on magnetic resonance imaging. ( **A**) Two-dimensional (2D) time-of-flight magnetic resonance angiography image shows roughly 50% stenosis of the carotid bulb resulting from a complex plaque. ( **B**) Contrast-enhanced long-axis T1-weighted (T1W) 2D double inversion recovery (DIR) image, obtained at a resolution of 0.35 × 0.35 × 2 mm. **(C)** Axial contrast-enhanced fat-suppressed T1W DIR image obtained with a resolution of 0.35 × 0.35 × 2 mm through the plaque reveals an enhancing fibrous cap (FC), a low-intensity non-enhancing lipid-rich necrotic core (LRNC), and a dark area of calcification. Images courtesy of Bruce Wasserman, Johns Hopkins University.

MRI protocols for carotid artery stenosis include sequences that quantify luminal stenosis—2D or 3D time of flight (TOF) magnetic resonance angiography (MRA) and contrast-enhanced MRA—and a set of sequences designed to identify IPH, the lipid-rich necrotic core (LRNC), plaque luminal surface ulceration, and intraplaque neovascularization. These MR examinations ideally should be performed on 1.5 or 3.0 Tesla scanners, preferably but not necessarily employing dedicated surface coils. Although a number of MR sequences are in clinical use, newer sequences share the ability to suppress signal from flowing blood to enable clearer visualization of the vessel wall and also to eliminate signal from fat, a feature that enables identification of the LRNC. Such sequences may be acquired using 2D (double inversion recovery T1 and T2) or preferably with 3D—magnetization-prepared rapid gradient echo (MPRAGE), multi-contrast atherosclerosis characterization (MATCH), or simultaneous non-contrast angiography and intraplaque hemorrhage (SNAP)—techniques ^21^. Three-dimensional techniques provide greater longitudinal coverage of the vessel. The reader is referred to Saba *et al*. ^20^ for a more comprehensive review of recommended MRI protocols. IPH typically demonstrates high signal on TOF, T1- and T2-weighted sequences, although this may vary depending on the age of the hemorrhage ( Figure 1). The LRNC is isointense on TOF images and hyperintense on T1- and T2-weighted images but, unlike IPH, demonstrates suppression of signal on fat-saturated sequences ^21^ ( Figure 2). Gadolinium contrast-enhanced sequences enable identification of plaque ulceration and neovascularization, both in the fibrous cap ( Figure 2) and in the shoulders and centrally within the plaque. Contrast-enhanced MRI is superior to unenhanced MRI in the characterization of fibrous cap thickness and also of the LRNC which appears as an area of poor to no enhancement ^22^ ( Figure 2). Dynamic contrast-enhanced (DCE) perfusion MRI can provide quantitative measures of plaque neovascularization with elevation of the volume transfer constant ( *K ^trans^*) in areas of high plaque neovascularity and inflammation. High-risk inflamed plaques also enhance on contrast-enhanced MR using ultra-small superparamagnetic iron oxide (USPIO), which are taken-up macrophages transformed from blood monocytes. Limitations for the routine use of MRI include long scanning times, presence of MR-incompatible devices, and risks associated with gadolinium-based contrast agent-related systemic toxicity and brain parenchymal deposition, especially in patients undergoing repeated examinations.

The presence of IPH on MRI also appears to be a potent tool for identifying risk of recurrent events. In a study of 179 patients, 63% were found to have evidence of IPH ^23^. In patients with more than 50% ICA stenosis and presence of IPH, the annual stroke rate was 23.2%. In patients with more than 50% stenosis and no IPH, the annual stroke was 0.6% ( *P* <0.001). Multi-center studies are needed to determine whether this technique should be a routine part of carotid imaging.

### Computed tomography

The reliability of CT angiogram (CTA) in the quantification of carotid stenosis and in delineating plaque surface characteristics such as ulceration is well established ^14, 15^. Newer dual-energy scanning technologies can precisely quantify calcification on contrast-enhanced CTA given the ability of this technique to differentiate calcium from iodine on the basis of the material-specific differences between the attenuations of x-rays of two energies. Plaques may be considered stable when at least 45% of their total volume is composed of calcium. Saba *et al*. recommend that CT for plaque imaging ideally be performed on third-generation scanners with at least 16 detector rows, 1-mm isotropic voxel resolution, and coverage extending from the aortic arch to the cranium ^24^. Such a protocol allows for Hounsfield units (HU)-based characterization of plaque components; values less than 60 HU are a feature of the LRNC, values between 60 and 130 HU imply mixed fatty and hemorrhagic components, and density greater than 130 HU is typical of calcification. Limitations of CTA include radiation exposure (especially in those patients who require repeated imaging), the need for administration of contrast medium (which carries risks of nephrotoxicity and anaphylaxis), and the inability to decisively depict IPH. The last of these, however, can be inferred from the presence of a “rim sign”, a rim of adventitial calcification encircling soft tissue density plaque, and from plaque surface ulceration IPH ^25^.

### Ultrasonography

B-mode US is a widely available tool that can provide quantification of carotid stenosis on the basis of luminal flow velocities; velocities greater than 230 cm/sec imply at least 70% stenosis to 99% stenosis ^11^. Criteria for near occlusion include a peak systolic velocity that is high, low, or undetectable, along with a variable result in the ICA-to-common carotid artery ratio.

Lipid-rich plaques are typically echolucent on US, whereas calcified plaques are hyperechoic and cast acoustic shadows. The latter, however, may also impede adequate visualization of the lumen and vessel wall. Contrast-enhanced US using microbubbles enables detection of plaque neovascularization ^26^. In a meta-analysis, Brinjikji *et al*. ^21^ recognized that a combination of features, including echolucency, neovascularization, ulceration, and plaque motion detected on US, are indicative of plaque complexity and hence may increase risk of future ischemic events, and the authors recommend that sonographic examination of carotid atherosclerotic disease must include a description of these features in addition to quantification of luminal stenosis. Detection of embolic signals using TCD is another modality to identify higher-risk patients.

### Positron emission tomography imaging

^18^F-fluorodeoxyglucose PET imaging may be used to characterize the severity of macrophage-driven plaque inflammation and to monitor response to treatment with statins and other novel agents. Newer agents such as ^18^F-fluorodeoxymanose (FDM), ^18^F-fluoromisonidazole (FMISO), and ^18^F-sodium fluoride ( ^18^F-NaF) hold promise in the characterization of macrophage subtypes, hypoxia, and calcification, respectively. PET imaging can be simultaneously performed with MRI on modern clinical PET-MR scanners and may herald a new era in plaque imaging where the exquisite quantitative measures derived from PET are combined with the soft tissue detail provided by MRI ^27^. At present, additional high-quality studies are needed before CEA/CAS can be advocated on the basis of high-risk plaque imaging features.

## Conclusions

Guidance on the optimal treatment of asymptomatic and symptomatic carotid stenosis continues to evolve. Clinicians must understand the impact of IMT in order to make informed decisions about which patients will benefit from revascularization ^28^. In addition, consideration of comorbid conditions and life expectancy is crucial in the evaluation of patients with asymptomatic disease. Advances in carotid plaque imaging may promise in the future to complement clinical developments to help optimize patient diagnosis and treatment.

The implications for symptomatic patients are similar: better outcomes with current medical treatment alone and fewer procedural indications. Measurements and risk stratification of outcomes with current optimal medical treatment alone are priorities for research in symptomatic and asymptomatic patients with ICA stenosis.
